# Controllable degradation kinetics of POSS nanoparticle-integrated poly(ε-caprolactone urea)urethane elastomers for tissue engineering applications

**DOI:** 10.1038/srep15040

**Published:** 2015-10-14

**Authors:** Lara Yildirimer, Asma Buanz, Simon Gaisford, Edward L. Malins, C. Remzi Becer, Naiem Moiemen, Gary M. Reynolds, Alexander M. Seifalian

**Affiliations:** 1 Centre for Nanotechnology & Regenerative Medicine, UCL Division of Surgery & Interventional Science, University College London; 2 UCL School of Pharmacy, University College London, 29-39 Brunswick Square, London WC1N 1AX, UK; 3 Department of Chemistry, University of Warwick, CV4 7AL, Coventry, UK; 4 School of Engineering and Materials Science, Queen Mary University of London, E1 4NS, London, UK; 5 Department of Burns and Plastic Surgery, University Hospitals Birmingham NHS Foundation Trust, Queen Elizabeth Hospital, Birmingham, United Kingdom; 6 The Healing Foundation Children's Burns Research Centre, University of Bristol, Senate House, Tyndall Avenue, Bristol, BS8 1TH, UK; 7 Centre for Liver Research & NIHR BRU, Queen Elizabeth Hospital & University of Birmingham; 8 Royal Free Hampstead NHS Trust Hospital, Pond Street, NW3 2QG, London, UK

## Abstract

Biodegradable elastomers are a popular choice for tissue engineering scaffolds, particularly in mechanically challenging settings (e.g. the skin). As the optimal rate of scaffold degradation depends on the tissue type to be regenerated, next-generation scaffolds must demonstrate tuneable degradation patterns. Previous investigations mainly focussed on the integration of more or less hydrolysable components to modulate degradation rates. In this study, however, the objective was to develop and synthesize a family of novel biodegradable polyurethanes (PUs) based on a poly(ε-caprolactone urea)urethane backbone integrating polyhedral oligomeric silsesquioxane (POSS-PCLU) with varying amounts of hard segments (24%, 28% and 33% (w/v)) in order to investigate the influence of hard segment chemistry on the degradation rate and profile. PUs lacking POSS nanoparticles served to prove the important function of POSS in maintaining the mechanical structures of the PU scaffolds before, during and after degradation. Mechanical testing of degraded samples revealed hard segment-dependent modulation of the materials’ viscoelastic properties, which was attributable to (i) degradation-induced changes in the PU crystallinity and (ii) either the presence or absence of POSS. In conclusion, this study presents a facile method of controlling degradation profiles of PU scaffolds used in tissue engineering applications.

Segmented polyurethanes (PUs) are amongst the most favoured synthetic polymers used in the manufacture of biomedical devices due to their superior biomechanical properties and a unique combination of toughness, durability, flexibility and biocompatibility. Generally, PUs are synthesized by reacting soft segments of polyesters or polycarbonates with hard segments of diisocyanates, which is followed by chain extension with diols or diamines^1^,^2^. Since their discovery by Otto Bayer in 1937, the PU family has become the most versatile of any family of plastic materials gaining considerable popularity in the biomedical field as pacemaker leads, catheters, vascular grafts and prosthetic heart valves^3^,^4^,^5^,^6^. More recently, a novel family of nanocomposite polyurethanes with polycarbonate soft segments and integrated polyhedral oligomeric silsesquioxanes (POSS) nanoparticles (POSS-PCU) has emerged as a clinically applicable non-resorbable tissue engineering scaffold with first-in-man transplantations of artificial tracheal replacements, vascular conduits, and lacrimal drainage systems^7^,^8^. Whilst non-resorbable scaffolds are adequately suited for certain applications, their lack of degradation limits their use to the adult population and potentially necessitates revision surgeries. Bioresorbable scaffolds, on the other hand, must maintain temporary structural integrity within relatively harsh wound environments and degrade in a controllable fashion surrounded by enzymes and free radicals, pH as well as temperature fluctuations. Numerous investigations have studied the biodegradation behaviour of segmented PUs by integrating more or less hydrolysable components such as polyethylene glycol (PEG), polylactic acid (PLA), and polyglycolic acid (PGA)^9^,^10^,^11^. However, degradation-induced loss of mechanical properties and rapid acidification of the degradation media limit their clinical application, particularly in mechanically demanding tissues such as the skin. Here, we have synthesized a novel POSS-integrated PU based on poly(ε-caprolactone urea)urethane with different ratios of hard segment (diisocyanate) content to obtain precisely tunable degradation rates.

## Results and Discussion

Biodegradation is influenced by both the chemical and physical properties of the PU; surface properties (e.g. surface area and wettability), first order structures (chemical structure, molecular weight and molecular weight distribution) as well as higher order structures (glass transition temperature, melting temperature, degree of crystallinity and elastic modulus) of polymers play fundamental roles in the biodegradation process^12^. It is well known that higher molecular weight PCL (*M*_*n*_ > 4000) is more slowly degraded by *Rhizopus delemar* lipase than that with low *M*_*n*_^13^. Similarly, polymer morphology, and by extension, their crystallinity affects degradation. Crystalline domains consist of tightly packed molecules, which are more resistant than amorphous regions where hydrolytic or enzymatic attack mainly occurs. Within the amorphous region, molecules are loosely packed making them more susceptible to degradation. Thus, degradation rate is inversely proportional to the crystallinity of the polymer^14^. Here, we have synthesized POSS-PCLU polymers of incrementally increasing hard segment content to evaluate whether or not the diisocyanate content may be a suitable parameter in the fine-tuning of polymer degradation. Polymers lacking POSS nanoparticles were also synthesized to determine the influence of POSS as a filler on degradation as previous studies have attributed an increased resistance to degradative breakdown of PUs when supplemented with POSS^15^. Degradation media spanning oxidative, hydrolytic and enzymatic conditions were selected to mimic as closely as possible the realistic environments encountered upon material implantation into the body. Under certain circumstances, *in vitro* degradation studies may even be considered superior to *in vivo* implantation studies as each individual degradative condition and their effects exerted onto the polymer may be studied separately. Implantable devices may encounter oxidative conditions as particularly leukocytes and macrophages involved in the early foreign body reaction are able to produce reactive oxygen species such as superoxides (O_2_^−^), hydrogen peroxide (H_2_O_2_), nitric oxide (NO) and hypochloric acid (HOCl)^16^,^17^. Particularly, aliphatic polyesters as used in the present study have been found to be susceptible to degradation via the nucleophilic attack of O_2_^−^ and subsequent cleavage of ester bonds^18^. This mechanism is believed to be a key component of the stress cracking phenomenon frequently observed in long-term implantable PU devices^19^. Water molecules can attack and break hydrolytically labile chemical bonds, breaking polymers into oligomers and finally monomers. Upon implantation, water can attack bonds located either on the surface or within the polymeric matrix via a water imbibition process. Since hydrolysis can be catalysed by acids or enzymes^17^, implantation of polyesters such as poly(lactic acid) or poly(glycolic acid) which have acidic degradation products, results in accelerated breakdown due to a process called autocatalysis. A further important factor influencing the mode and rate of degradation is the polymer’s surface wettability with hydrophilic materials degrading more readily than hydrophobic ones. In terms of PUs, hard segment content as well as distribution within the amorphous region are postulated to significantly influence hydrolytic degradation as hydrolysable bonds may be masked by compact hard segment micro-domains, which can form by hydrogen bonding between individual hard segments. Enzymes are cell-derived proteins capable of accelerating hydrolytic degradation processes. Naturally occurring and principally important in the breakdown of substrates for facilitated nutrient uptake, enzymes have also been demonstrated to accelerate biomaterial degradation^20^. The content and distribution of hard segments within the polymeric surface was shown to influence the manner in which enzymes adsorb and express their activity on the material’s surface so that increasing hard segment contents correlated with reduced degradation^21^,^22^.

### Synthesis and molecular characterization of non-degraded POSS-PCLU films

**Figure 1:**
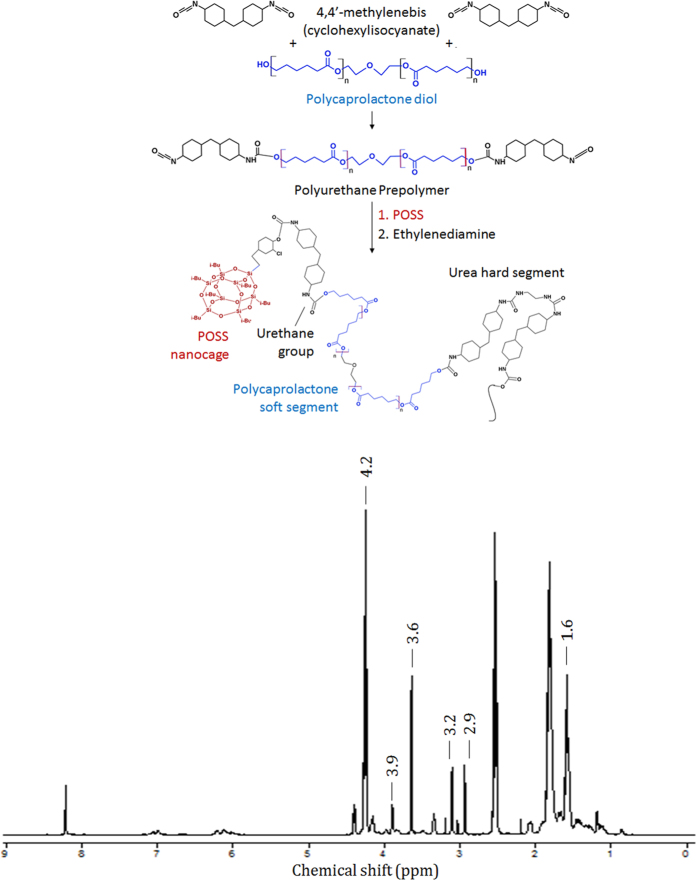
(**a**) Synthesis of the aliphatic poly(ε-caprolactone urea)urethane prepolymer in a 2:1 ratio of 4,4′-methylenebis (cyclohexylisocyanate) to poly(ε -caprolactone)diol. Chain extension reaction of the poly(ε-caprolactone urea)urethane prepolymer with ethylenediamine and integration of POSS nanoparticles as endcapped pendant chains. The final segmented polyurethane consists of urea hard segments linked to poly(ε-caprolactone) soft segment by urethane bonds. (**b)** ^1^H nuclear magnetic resonance (NMR) spectrum of a representative POSS-PCLU polyurethane.

All PUs were synthesized from polycaprolactone diol with different dicyclohexylmethane diisocyanate contents, i.e. 24%, 28% and 33%, using an ethylenediamine chain extender (Fig. 1a). The compositions are defined in Table 1. The chemical structures of POSS-PCLU were verified by ^1^H NMR spectroscopy (Fig. 1b). The range of hard segment content was as stated since below 10%, hard segments have been demonstrated to offer only insufficient crosslinking capacity resulting in poor mechanical performance^23^. Too high a hard segment content, on the other hand, significantly interferes with hydrolytic or oxidative degradation due to stearic hindrance of chain scission sites by H-bonds. Prior to degradation, synthesized PUs of different hard segment (HS) content exhibited similar FTIR features but subtle NMR differences at 1.6 ppm (results not shown). Such similarities are to be expected considering that PCLU is the major component. However, the small spectroscopic variations observed are congruent with the use of different concentrations of hard segment. Soft and hard segment *T*_*g*_ of all non-degraded polymers were in the range of −52.9 °C to −55.5 °C and +40.8 °C to +53.3 °C, respectively. Table 2 summarizes the number average molecular weights (*M*_*n*_) and molecular weight distributions (polydispersity index, PDI) of all variations of the PU before and after degradation in each buffer for a maximum period of 6 months. The symmetrical and narrow bell shaped molecular weight distribution curve of a control non-degraded PU demonstrates successful and complete polymerization (supplementary Fig. 1). The molecular weights of these controls ranged from 11.5 × 10^4^ g/mol to 11.9 × 10^4^ g/mol with their corresponding molecular weight distributions between 1.4 and 1.7. The *M*_*n*_ decreased with increasing hard segment content due to a corresponding decrease in the proportion of PCL, which is the highest *M*_*n*_ component in the PUs. Addition of POSS nanoparticles resulted in a higher *M*_*n*_ compared to PCLU-24 (24% hard segment content) without POSS nanoparticles due to the additional molecules (POSS *M*_*w*_ ~1kDa). Contrary to expectations, no significant trend could be observed between POSS inclusion and *M*_*n*_ post-degradation indicating too small a fraction of POSS (2%).

### Changes in Mw after degradation

**Figure 2:**
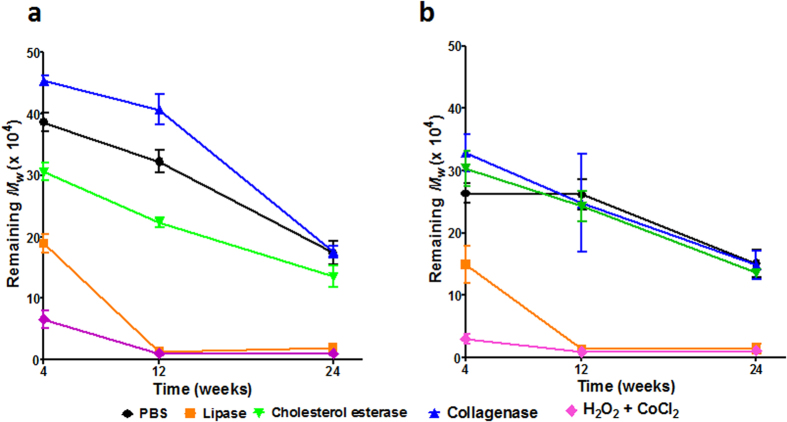
Molecular weight loss over time after degradation in PBS, lipase, collagenase, cholesterol esterase and hydrogen peroxide buffers. (**a**) PCLU-24 and (**b**) POSS-PCLU-24. Degraded samples of POSS-PCLU-28 and POSS-PCLU-33 could not be sufficiently dissolved in solvent and data is therefore missing. Values are shown as mean ± 1 SD.

Exposure to lipase and hydrogen peroxide buffers resulted in the most significant mass losses, highest PDI and relatively higher crystallinities, consistent with previous reports^24^,^25^,^26^. Increasing the hard segment content predictably resulted in better resistance to degradation as demonstrated by incrementally decreasing crystallinities coupled to higher molecular weights (Table 2). POSS nanoparticle addition, however, did not seem to induce any changes in degradation behaviour as postulated by Mather group^15^. This discrepancy is likely due to the significantly smaller percentage fraction of POSS integrated into our polymeric systems (2% versus 20%). Figure 2 demonstrates a clear time-dependent reduction of *M*_*w*_ for PCLU-24 and POSS-PCLU-24 degraded in different degradation media. At the final time point of this study (6 months in buffer solutions), PUs exposed to lipase and hydrogen peroxide demonstrated the most significant reductions in *M*_*w*_ of >90%. This is consistent with a combination of general bulk degradation coupled to some enzymatic surface erosion^27^. Exposure to PBS for 6 months resulted in only minor degradation (<15%) suggesting limited hydrolytic scission of water-labile ester linkages. Extensive hydrogen-bonding between hard segment micro-domains has previously been suggested to mask cleavage sites, thus retarding degradation^22^. Similarly, bathing in collagenase buffer for 6 months resulted in less severe degradation compared to exposure to lipase and hydrogen peroxide buffers. A maximum loss in *M*_*n*_ of <25% was evident for collagenase-degraded PUs. Again, correlations between hard segment content and mass loss were evident. This oft-suggested theory of hard segment content-dependent protective micro-domain formation is also reflected in ATR-FTIR and DSC results (see below).

### Surface Changes Induced by Degradation

**Figure 3:**
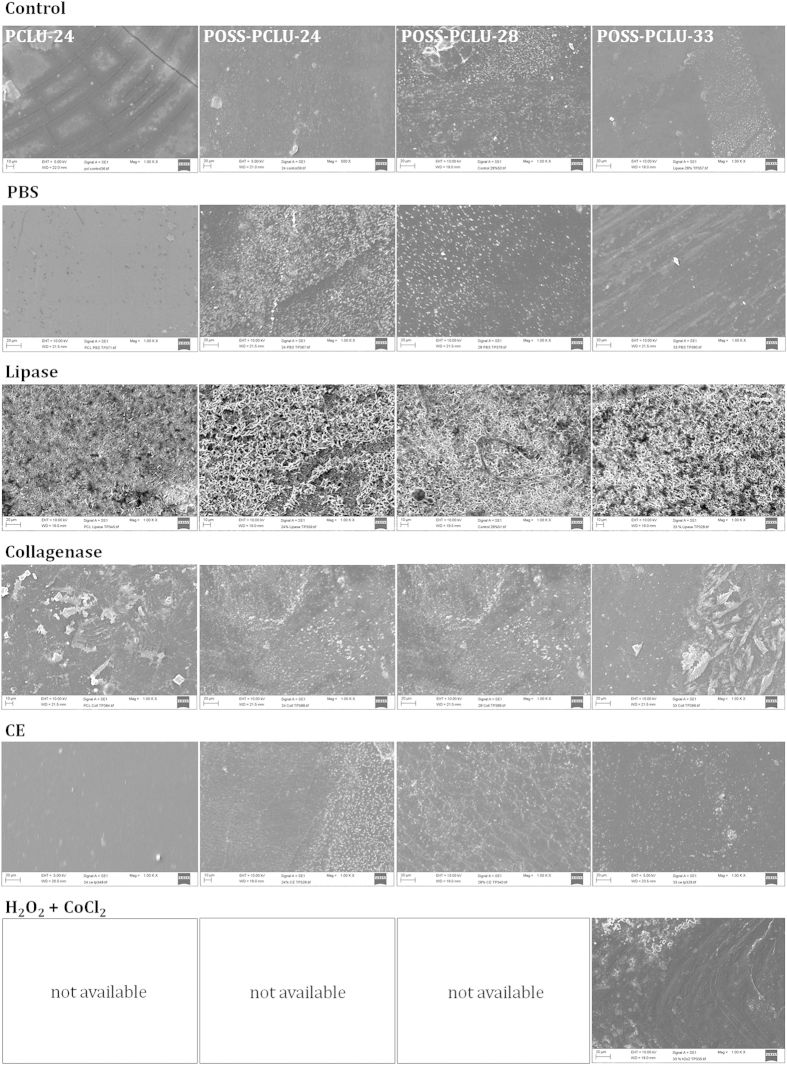
Scanning electron microscopy images of control and degraded polymers incubated in buffers for 6 months.

**Figure 4:**
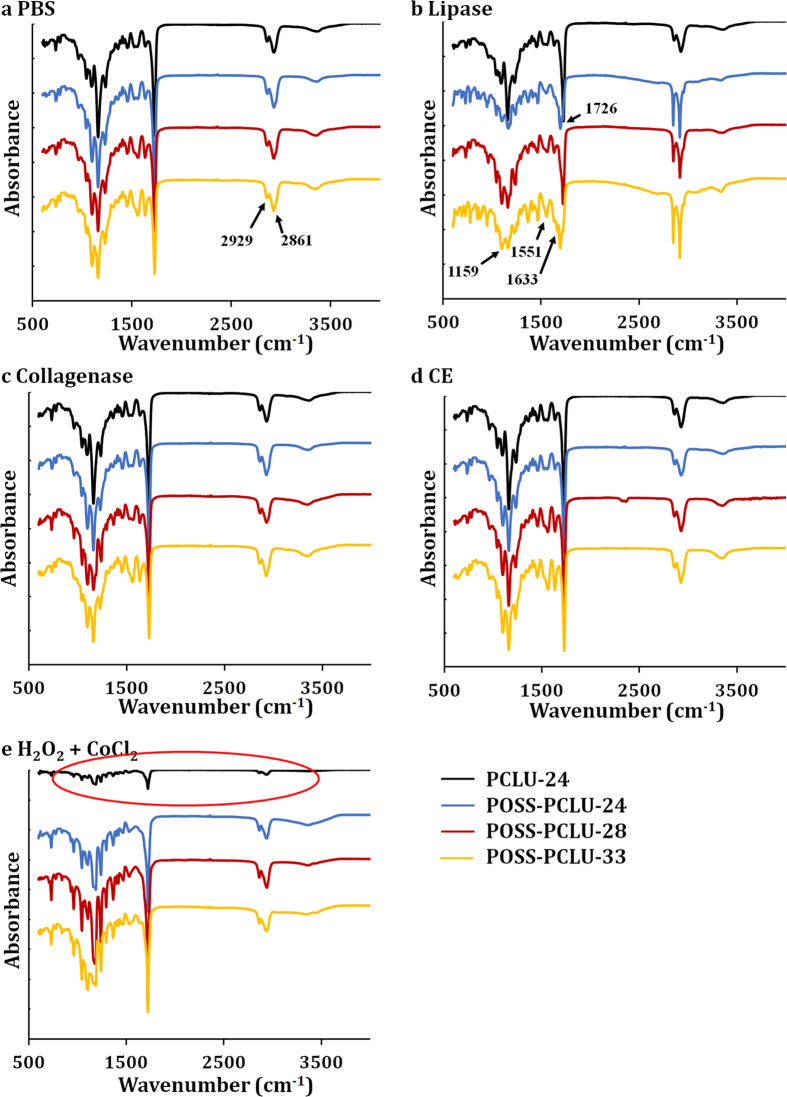
Overlaid attenuated total reflectance (ATR)-Fourier transform infrared (FTIR) spectra of films after 6 months in different buffers.

Scanning electron microscopy (SEM) images show degradation-induced surface changes of control and degraded PUs after 6 months in buffer solutions (Fig. 3). All control, non-degraded films except PCLU-24 revealed spherical micro-particulates on their surfaces which we hypothesize can be assigned to micro-aggregates of POSS nanoparticles as they cannot be seen in films lacking POSS^28^. Compared to control films, all degradation buffers induced surface changes in the tested PUs. All films incubated in lipase buffer displayed uniform roughened and porous surfaces, indicating a surface-erosion mechanisms which is typical of enzymatic attack^29^. Such gradual degradation from non-porous into porous films potentially enables these PU scaffolds to be used as delivery vehicles for a multitude of molecules such as drugs or genes^30^. Films exposed to cholesterol esterase and hydrogen peroxide buffer, on the other hand, revealed significant stress-cracking associated with degradation. This is of clinical significance as inflammatory cells including monocyte-macrophages release both cholesterol esterases and oxidants capable of degrading PUs^21^,^23^. Overall, most degradative changes could be observed in films exposed to lipase and hydrogen peroxide buffers which is in agreement with results obtained by GPC and mechanical characterization. Contact angle measurements were conducted to evaluate polymer wettability as hydrophilicity is known to be an important parameter in hydrolytic degradation as well as cellular adhesion. Hydrolysis requires the reaction between water and labile ester bonds and the reaction velocity depends on the abundance of both water and ester bonds. Thus, hydrophilic polymers capable of attracting water molecules generally degrade more rapidly than hydrophobic polymers presuming the ester bond concentration remains relatively stable^31^. Control POSS-PCLU polymers did not exhibit significant differences in contact angle (*θ*) with a mean hydrophobic *θ* of 105°. PUs lacking POSS nanoparticles predictably were more hydrophilic as POSS is known to increase hydrophobicity when incorporated into polymeric systems^32^. Upon degradation, contact angles were shown to gradually decrease, in line with expectations as shown in Table 2. As degradation progresses, the PCL component is preferentially broken down due to hydrolytically labile ester bonds. A reduced amorphous soft segment component thus favours relatively increased phase mixing which results in the polar hard segments aggregating at the material surface. This, in turn, reduces contact angle. It can further be observed that with higher initial hard segment content, degradation-induced increases in material hydrophilicity are more pronounced due to the relatively more polar segments within the degraded material. ATR-FTIR spectra of PCLU-24 and POSS-PCLU-24, POSS-PCLU-28 and POSS-PCLU-33 degraded in different buffers were overlaid for comparison (Fig. 4). All PUs exhibited common peaks at 2929 cm^−1^ and 2861 cm^−1^ which correspond to the alkyl groups of the hard segment, HDI. The peaks at 1726 cm^−1^ and 1159 cm^−1^ represent the carbonyl (C = O) region of non-hydrogen-bonded urethane and the ester (O-C-O) absorbance of the soft segment, respectively. The heights of the latter two peaks are negatively correlated with increasing hard segment content. In the case of the C = O bond of the non-hydrogen-bonded urethane bonds, the lower height with higher percentage hard segment is due to the fact that in high hard segment polymers, most urethane bonds are hydrogen-bonded, leaving a smaller fraction of urethane bonds non-bonded. With increasing hard segment content, the spectral peaks corresponding to hydrogen-bonded urethane (1633 cm^−1^) and urea (1551 cm^−1^) bonds increase in height providing a qualitative measure of bond content. Both PCLU-24 and POSS-PCLU-24 containing the lowest amount of hard segment demonstrate relatively smaller peaks compared to the polymers of higher hard segment content. Similarly, polymers containing POSS nanoparticles exhibit a high peak at around 1094 cm^−1^ whilst PCLU-24 does not. After degradation in lipase buffer, and particularly in hydrogen peroxide buffer, significant loss of peak height at 1159 cm^−1^ and 1726 cm^−1^ was observed, indicating a breakdown in soft segment. PCLU-24 was noted to suffer loss of peak height of all major peaks after degradation in peroxide (Fig. 4e, circled red). Hard segment breakdown was also noted when exposed to lipase and hydrogen peroxide with a loss in peak height at 1633 cm^−1^.

### Thermal Properties of Degraded POSS-PCLU

**Figure 5:**
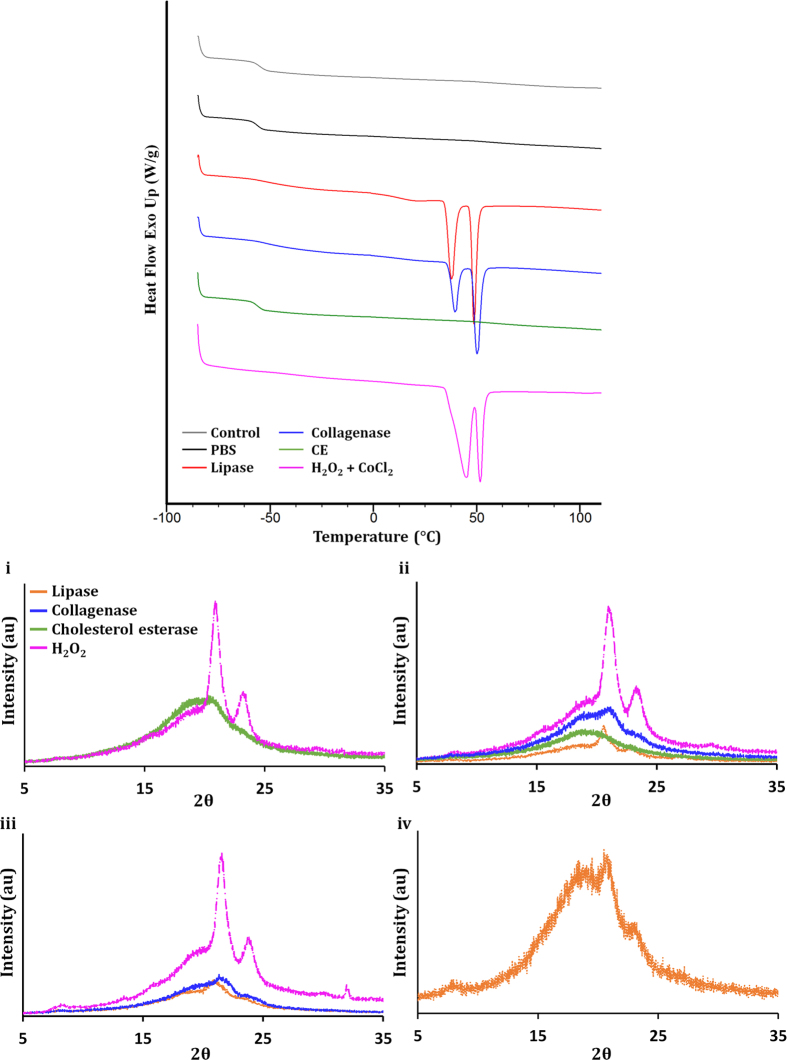
(**a**) Representative differential scanning calorimetry graph of the polymer after 6 months of incubation. (**b**) XRD patterns of degraded (i) PCLU-24, (ii) POSS-PCLU-24, (iii) POSS-PCLU-28 and (iv) POSS-PCLU-33. Degradation time was 6 months.

Segmented PUs display several thermal transitions, corresponding to the microstructure of the soft and hard domains. The thermal properties of cast control and degraded POSS-PCLU films with different percentage hard segments are summarized in Table 2. In general, soft segment glass transition temperatures, *T*_*g*_, of POSS-PCLU and PCLU were independent of the hard segment content (ranging from 24%–33%) indicating limited hard segment mixing within the soft domain. Similar results were found with PU systems based on poly(ethylene oxide), poly(tetramethylene oxide) and poly(propylene oxide) with comparable hard segment contents^33^,^34^,^35^. *T*_*g*_ of the PCLU soft segments ranged from approximately −60.0 °C for the control, non-degraded sample to −25 °C after oxidative degradation. The *T*_*g*_ shift to higher temperatures induced by the different degradation media indicates a degree of phase mixing between the soft and hard segment of the PU and crystallization of amorphous areas^36^. Oxidative conditions were seen to induce the most drastic increase in *T*_*g*_, particularly in samples of relatively lower hard segment content, suggesting strong interactions between soft and hard segments secondary to degradation. Previous studies have confirmed the formation of hard segment micro-domain structures in segmented PUs which can protect or mask potential cleavage sites within their hard segments (i.e. urea and urethane bonds) via formation of an extensive network of hydrogen bonds (H-bonds)^37^,^38^. Thus, with increasing hard segment content, the extent of urethane H-bonding also increases, resulting in delayed degradation. This protective phenomenon was, indeed, present in the samples with relatively higher hard segment content which resisted a significant increase in *T*_*g*_ post-degradation. None of the control polymers exhibited hard segment crystallization and hard segment *T*_*g*_ ranged from approximately 40 °C to 55 °C which is just below that observed by Skarja *et al.*^36^. The presence of a glass transition means that the hard segments of all control polymers are amorphous at room or body temperature^36^,^39^ which may be due to a lack of efficient chain packing necessary for hard segment crystallization^40^ secondary to the mobile aliphatic diisocyanate^41^ and the presence of bulky side-chains on the chain extender (ethylene diamine). Endothermic melting peaks were observed in samples exposed to lipase, collagenase, cholesterol esterase and hydrogen peroxide between 35 °C and 50 °C which were attributed to the melting of the crystalline portions of the soft segment^42^. Double endotherms could be observed in all films exposed to lipase and hydrogen peroxide (Fig. 5a) whilst no endothermic peaks were seen in control and PBS exposed samples, indicative of a mostly amorphous starting polymer and negligible degradation in PBS-exposed samples. Collagenase exposure induced the formation of a double melting peak only in POSS-PCLU-28 after 6 months and a small single endothermic peak in POSS-PCLU-24 after the same exposure period. Cholesterol esterase exposure did not induce melting peaks in any of the samples except PCLU-24 where a single small endothermic valley could be observed after 6 months of exposure. Double endothermic peaks may indicate the presence of either two distinct types of crystals or the recrystallization of a metastable crystal into a more stable form with increasing temperatures within polymorphic structures. The latter underlies the principles of Ostwald’s rule of stages, which states that when a material crystallises, it will move to equilibrium from an initial high-energy state (e.g. glass) via a cascade of stages requiring minimal changes in free energy. This implies that the sample will crystallise in sequence beginning with the least stable polymorph and progressing through any further metastable polymorph to the stable crystalline form^43^. The melting temperature and percentage crystallinity are affected by the soft segment content and its molecular weight^12^. After oxidative and enzymatic degradation with hydrogen peroxide and lipase, respectively, the degree of crystallinity was positively correlated with the relative amounts of soft segments in the study samples, such that percentage crystallinity followed: PCLU-24 > POSS-PCLU-24 > POSS-PCLU-28 > POSS-PCLU-33. The higher the hard segment content within the samples, the lower the relative soft, crystallizable portion, which is reflected in the DSC thermograms. Additionally, increases in soft segment *T*_*g*_ were correlated with higher percentage crystallinity induced by degradation (supplementary Figure 2) and increased crystallinity resulted in a reduction in *T*_*g*_ step height secondary to a reduction in the amorphous content (supplementary Figure 3). This is likely due to increased phase mixing secondary to shorter chain length and thus reduced molecular weight of the soft segment. For reference, the crystal melting temperature for pure PCL is 57 °C^44^ which was higher than those observed in the degraded samples, suggestive of phase mixing. A small exothermic transition was detected towards the lower temperature side of the main endothermic peaks in all those samples exhibiting melting peaks and was attributed to the crystallization transition (*T*_*c*_). XRD studies on films exposed to oxidative medium for the total period of 6 months revealed PCL peaks at 2θ = 21.3° and 23.7° for all PUs, in accordance with previous studies^45^. Similarly, exposure to lipase and collagenase buffers resulted in these PCL peaks, albeit at lower intensities (Fig. 5b). This so-called peak splitting suggests the presence of different crystalline domains within some degraded films^46^. This hypothesis is corroborated by the presence of double endothermic melting peaks on DSC traces of those samples which display peak splitting on XRD. Diffractograms of films degraded in hydrogen peroxide revealed further peaks at 2θ = 8° and 29.6° as well as broad shoulders at 15.7° and 19.2°. Neither cholesterol esterase buffer nor PBS incubation revealed peaks, suggestive of a lesser degree of degradation and relative conservation of the amorphous PU structure. Comparisons between different time points indicate a time-dependent formation of crystal structures, which can also be observed in the DSC traces. These results suggest a condition- and time-dependent breakdown of the mostly amorphous PU into semicrystalline structures secondary to degradation-induced changes.

### Degradation-Induced Changes in Mechanical Properties

**Figure 6:**
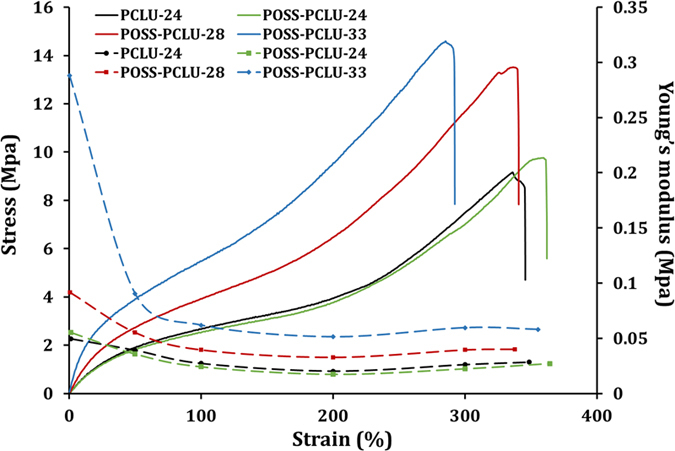
Representative stress-strain curves (—) overlaid against Young’s moduli (—) for PCLU-24, POSS-PCLU-24, POSS-PCLU-28 and POSS-PCLU-33. Degradation time was 6 months.

Degradation of PU films resulted in significant changes in mechanical properties (Table 2), contrary to previously published reports^15^. Differences in outcomes may be attributable to major differences in incubation periods between different studies with short time spans (days rather than months) failing to induce changes and thus potentially misrepresenting long-term properties of investigated materials. In general, films incubated in any degradation buffer exhibited higher values for stress and strain at break, indicative of increased phase separation induced by degradation. Representative stress-strain curves for all control materials are shown in Fig. 6. Overlaid on those graphs are the Young’s moduli, a measure for material stiffness. Specific degradation points (1, 4 and 6 months) were chosen for mechanical evaluation but even after 4 weeks, PCLU-24 incubated in lipase or hydrogen peroxide buffer disintegrated, making tensile testing impossible. In general, both tensile strength and Young’s modulus increased with increasing hard segment content for control non-degraded films, which is likely due to increased crystalline to amorphous content ratio within the PUs leading to better phase separation as well as urethane bond interactions^47^. Films incubated in collagenase buffer for 6 months exhibited the largest increase in both Young’s modulus and tensile strength whilst retaining dependence on the hard segment content as observed for non-degraded control films (films exposed to lipase buffer or hydrogen peroxide became too brittle to handle after 6 months and thus were not included for mechanical analysis). This suggests a degradation-induced influence on these mechanical parameters. However, films not containing any POSS nanoparticles (PCLU-24) demonstrated the highest increase in Young’s modulus compared to their compositional equivalent containing POSS (POSS-PCLU-24). This suggests a major role for POSS in protecting PUs from degradation induced loss in material elasticity and structure. This is important in the development of 3D scaffolds for tissue engineering applications; throughout the initial stages of degradation, the scaffold may maintain its structural integrity while gradually freeing up space for tissue infiltration. Cholesterol esterase buffer did not significantly impact upon the Young’s modulus after 6 months’ of incubation, however, a negative trend in modulus after PBS exposure could be observed for all PU films. This is likely due to a lack in phase mixing which is usually expected during degradation. Thus, significant phase separation results in dispersion of the crystalline hard segment within the amorphous region of the PU as supported by the lack of crystallinity data obtained using DSC spectral results. Small Young’s moduli are generally believed to suffer from high strain accumulation resulting in poor durability, thus compromising a material’s *in vivo* stability^48^.

### Cytocompatibility of Degradation Products

**Figure 7:**
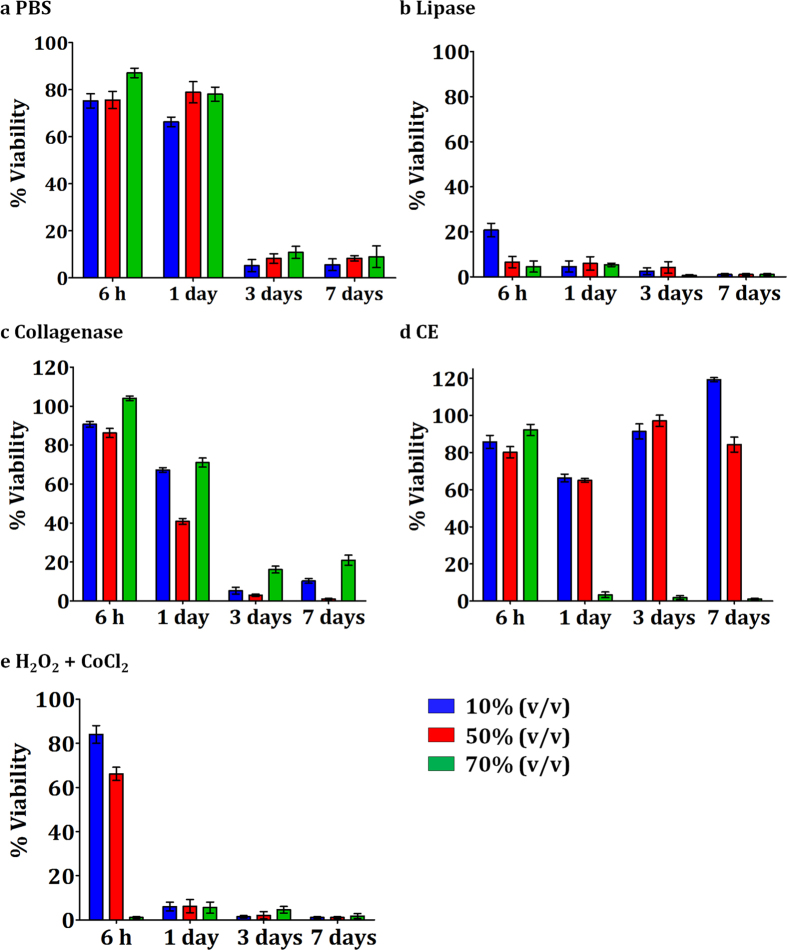
AlamarBlue® cell viability assay over a period of seven days. HDFa were cultured in media containing extracts of polymers degraded in degradation buffers for 6 months. Increasing concentrations of extracts represent the *in vivo* scenario where highest extract concentrations can be found in close proximity to the polymer scaffold.

In general, a time- and concentration-dependent decrease in cellular viability was observed when HDFa were incubated with degradation extracts of any PU (Fig. 7). In addition, toxicities seemed to be related to the hard segment content in the PUs. Higher hard segment PUs translated into lower cell viability. The results of this toxicity study were unexpected as theoretically, no component of the POSS-PCLU nanocomposite polymer is toxic individually^49^. Similarly, HDI as opposed to MDI hard segment was utilized due to its supposed superior biocompatibility^50^. Most interestingly, previous studies using similar PU compositions to the one currently under investigation reported cytocompatibility and non-toxicity^51^. One likely reason may be that the majority of toxicity studies are carried out by directly growing cells on polymer surfaces rather than in a medium containing their degradation extracts. Also, *in vivo* subcutaneous implantation studies of PCLU and POSS-PCLU variations revealed no significant inflammatory response (see below). We hypothesize that acidic degradation products were responsible for cell death rather than true cytotoxicity of the metabolites^52^. Taken together, these cytocompatibility results, whilst indicative of potential toxicity, may not be extrapolated directly into an *in vivo* setting as the body’s circulatory potential is expected to flush away materials, which may cause harm at high concentrations. Here, we used volume percent concentrations of degradation extracts to simulate highest concentrations of leachables closest to the implanted PU scaffold with gradually decreasing concentrations further away. One limitation of this approach appears to be the incrementally decreasing percentage of growth medium, which may be an explanation as to the concentration-dependent decrease in cell viability. More realistic means of analysis, therefore, include *in vivo* exposure studies over a sufficient time period.

### *In Vivo* microvascular blood flow and oxygenation within 3D scaffolds

**Figure 8:**
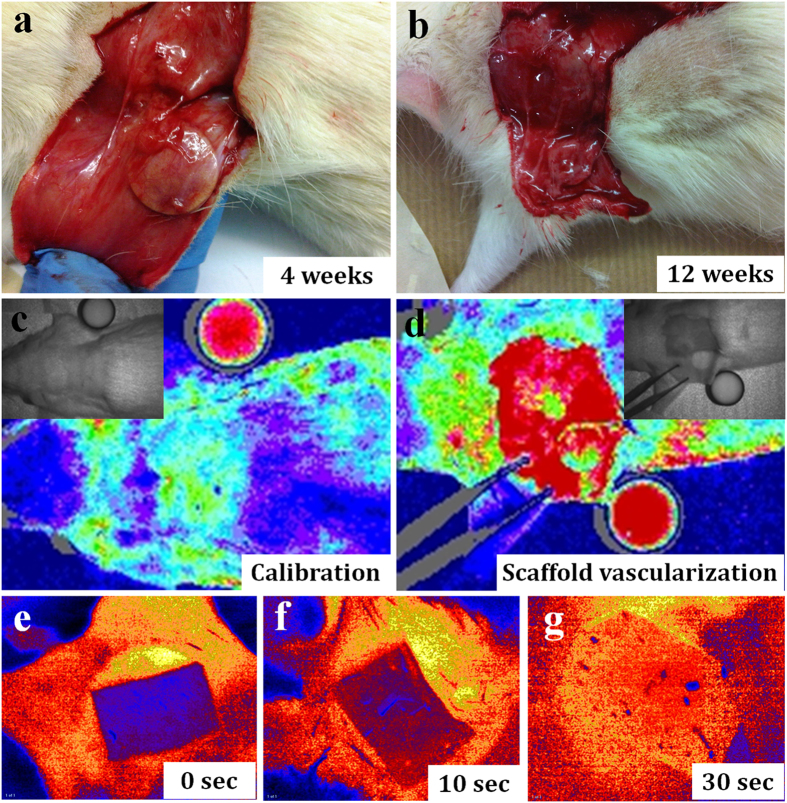
Macroscopic view of subcutaneously implanted scaffolds at (a) 4 weeks and (b) 12 weeks. (**c**) Laser Doppler calibration using full fat milk and (**d**) analysis of vascularized scaffolds prior to explantation. (**e**–**g**) Monitoring of oxygen transfer from scaffolds through an oxygen sensor film as a measure of scaffold vascularization.

Subcutaneous integration and the extent of vascularization were assessed visually, using a laser Doppler perfusion imager and an oxygen sensor (Fig. 8). Animals were anaesthetized and scaffolds were exposed. Visual comparison between scaffolds of different hard segment and different lengths of implantation revealed obvious dimensional differences. POSS-PCLU-33 scaffolds implanted for the shortest period (4 weeks) appeared to retain most of their size compared to PCLU-24 scaffolds implanted for 12 weeks, which were smallest. All scaffolds, however, appeared to be firmly integrated with the subcutaneous tissue and encapsulated in a highly vascularized fibrous sheath. Qualitative measurements of the extent of superficial scaffold vascularization were obtained using laser Doppler perfusion imaging and oxygen sensing, the premise being that vascularized scaffolds would be able to carry oxygen to the scaffold surface, which could be detected by a sensor foil placed onto the scaffold. The intensity of the change in colour of the sensor foil determined the extent of vascularization. Whilst relatively non-specific as a stand-alone analytic tool, combined with histological and immunohistochemical analyses, the ability and extent of vascularization may be confidently determined.

### Histological and Immunohistochemical Analysis of Explanted Samples

**Figure 9:**
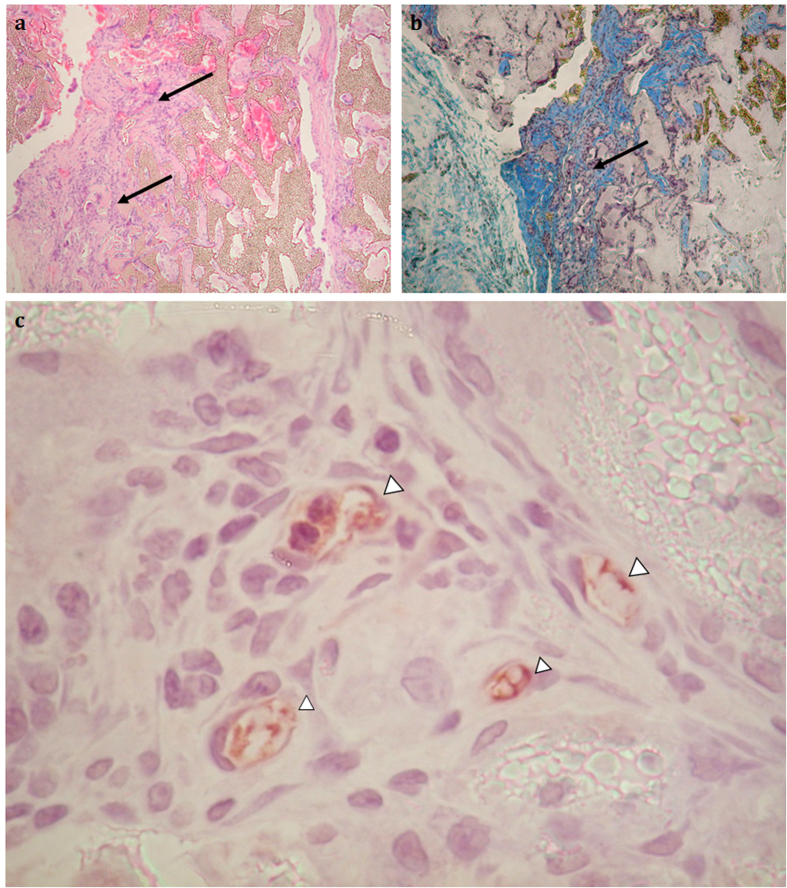
Haematoxylin & eosin staining of subcutaneously implanted scaffolds after 8 weeks and corresponding MSB stained image. The arrows indicate noticeable integration of the tissue infiltrate with the scaffold (**a**) and marked collagen deposition (**b**). (**c**) Vascular infiltration visible at 4 weeks post-implantation.

Since biodegradable regeneration scaffolds will be used *in vivo*, the potential tissue-polymer interactions, local and systemic toxicities require evaluation. Thus, histological sections of the implant and surrounding tissues as well as all major end organs were analysed for signs of potential toxicity. Gross visual analysis during autopsy revealed a thin fibrous sheath encapsulating all scaffolds. H & E staining of implanted PCLU-24 scaffolds showed minimal foreign body giant cell infiltration whilst none of the POSS-PCLU scaffolds revealed any signs of sustained inflammation, indicating that the addition of POSS nanoparticles rendered the scaffold more immune tolerant. Generally, it is well known that polycaprolactone has good biocompatibility and its degradation product, 6-hydroxyhexanoic acid, is transformed by microsomalu-oxidation to adipic acid, a natural occurring metabolite^53^. At 12 weeks post-implantation, gross evaluation of explanted scaffolds and surrounding tissues revealed no signs of infection. All scaffolds supported cellular infiltration and collagen deposition. In general, scaffolds based on lower hard segment content PUs experienced significantly more tissue infiltration compared to higher hard segment content scaffolds. This is likely due to increased degradation of softer polymers. Arguably, PCLU-24 was more degraded compared to its POSS-containing counterpart, supporting the hypothesis of POSS-induced slowing of degradation. Some polymers folded up on themselves despite the central suture. This, however, did not result in brittle fracture, underlining the elastic nature of these scaffolds. Histological examination of the polymer sections (H&E) demonstrated that each PU group supported cellular infiltration and connective tissue ingrowth which traversed from the periphery of the polymer scaffold through to the opposite side. Cells initially populated the pores of the scaffolds before breaking through the polymeric structures to infiltrate the scaffold proper. Over time, collagen deposition and vascular infiltration increased which was accompanied by time- and hard segment content-dependent degradation of scaffolds (Fig. 9a,b). At 4 weeks post-implantation, 50% of the PCLU-24 graft was populated with cells and a moderate amount of vasculature and collagen formation. At 12 weeks post-implantation, collagen deposition was more abundant. Similar trends were observed for POSS-PCLU-24 scaffolds. However, more abundant collagen deposition and vascular infiltration was noticed at the 12 week time point (Fig. 9c). Scaffolds of higher hard segment content initially revealed significantly less cellular infiltration compared to softer scaffolds but demonstrated cellular proliferation rates similar to those observed in the softer scaffolds, indicating slower degradation of the PU scaffolds and thus slower tissue infiltration. Histological sections of end organs revealed no signs of inflammation when exposed to degradation products of any of the scaffolds (supplementary Figure 4), suggesting excellent biocompatibility of both PCLU and POSS-PCLU scaffolds.

## Conclusion

We have developed and synthesized a novel nanocomposite PU based on a poly(ε-caprolactone urea)urethane backbone integrating POSS nanoparticles as side chains. Differences in hard segment content and presence or absence of POSS particles were analysed with respect to their influence on *in vitro* and *in vivo* degradative behaviour. Degradation was strongly dependent upon hard segment micro-domain formation with PUs containing the highest number of hydrolytically labile urea and urethane bonds exhibiting the least degradation. Additionally, the mode of degradation was strongly influenced by POSS nanoparticle inclusion due to its ability to influence degradation. Rather than degrading into a stiff and brittle film which may compromise functionality of the implanted device, POSS nanoparticles were able to tailor material degradation whilst retaining vital mechanical structures and elasticity. This is of great importance for tissue engineering applications in development of organs involving tissues such as the skin or vasculatures.

## Methods

### Polymer Synthesis

The polymer was prepared according to a previously published method^54^. In brief, dry polycaprolactone diol (*M*_*w*_ 2000) and *trans*-cyclohexanechloroydrinisobutyl-POSS were placed in a reaction flask equipped with a mechanical stirrer and nitrogen inlet. The mixture was heated to 135 °C to dissolve the POSS nanocage and then cooled to 60 °C. To this, dicyclohexylmethane diisocyanate (Desmodur W, Bayer) was added and reacted under nitrogen at 70 °C for 90 minutes to form a pre-polymer. The molar ratio of terminal NCO to (OH + NH_2_) was 1:1. Then, *N,N*-dimethylacetamide (DMAc) was added and the mixture cooled to 40 °C. A mixture of ethylenediamine and diethylamine in DMAC was added to allow chain extension of the pre-polymer. 1-Butanol in DMAc was added to the mixture to form an 18% polyhedral oligomeric silsesquioxane-poly(ε-caprolactone urea)urethane (POSS-PCLU) solution. Polymers of three different percentage hard segments were synthesized by varying the amount of dicyclohexylmethane diisocyanate to obtain polyurethanes of 24%, 28%, and 33% hard segment. The hard segment content was calculated as the sum of chain extender and the weight of diisocyanate as a percentage of the total weight. A control lacking POSS nanoparticles was synthesized to analyse the influence of silica nanoparticles on (i) *in vitro* cytocompatibility, (ii) *in vivo* biocompatibility, and (ii) any potential inhibitory effect of POSS nanoparticles on degradation. The polymer nomenclature was based on their main constituents and the weight percentage hard segment, as POSS-PCLU-24, POSS-PCLU-28, POSS-PCLU-33, and PCLU-24. All chemicals and reagents were purchased from Sigma Aldrich Ltd. (Gillingham, UK). Figure 1 schematically summarises the reaction steps.

### Scaffold Fabrication

Samples were fabricated by either casting or coagulation/phase inversion method to obtain non-porous films or porous sponges, respectively. Cast sheets were prepared by pouring diluted 15 wt.% POSS-PCLU polymer solution (~ 8 mg) into a clean Petri dish (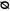 10 cm) and allowing solvent evaporation overnight at constant temperature (65 °C). Coagulated scaffolds were prepared by a sacrificial porogen leaching technique combined with a phase inversion coagulation method. The polymer solution was supplemented with sodium bicarbonate (NaHCO_3_) particles (Brunner Mond, Cheshire, UK) and surfactant (Tween 20) to obtain viscous slurry. This was poured over a surface modified stainless steel plate. Top surface modification was applied to avoid skin formation and increase percentage porosity. The polymer mixture was then extruded according to a protocol developed in-house. Overnight extrusion in sterile de-ionized water resulted in solvent exchange and NaHCO_3_ leaching and the fabrication of porous scaffolds. Washing over a period of 48 h using regular changes of sterile de-ionized water completely removed all remaining salt particles and DMAc, as confirmed by inductively coupled plasma optical emission spectrometry (Warwick Analytical Services, Coventry, UK).

### *In Vitro* Degradation

Accelerated degradation experiments were carried out according to ISO 10993:12 specification under hydrolytic, enzymatic and oxidative conditions at 37 °C. Cast specimen of the different polymers were immersed in 5 mL of (i) sterile PBS, (ii) lipase (10 U/mL), (iii) collagenase (10 U/mL), (iv), cholesterol esterase (1 U/mL), or (v) 20% H_2_O_2_ in 0.1 M cobalt chloride (CoCl_2_). Degradation media were changed every week to replenish enzymatic/oxidative activities and supernatants containing the degradation products were collected for subsequent toxicological analysis. At selected time points, specimen were washed thrice with sterile distilled water and the air-dried overnight before being subjected to analyses.

### Evaluation of *In Vitro* Degraded Films

#### ^1^H Nuclear Magnetic Resonance (^1^H NMR) Spectroscopy

The chemical structure and composition of all 4 PUs were determined by ^1^H NMR spectroscopy using a Bruker AV 400 NMR spectrometer. The NMR spectrum was obtained at room temperature in deuterated dimethyformamide (DMF_*d*_) (50 mg/mL) with tetramethylsilane (TMS) as an internal standard.

#### Attenuated Total Reflectance (ATR)-Fourier Transform Infrared (FTIR) Spectroscopy

Fourier Transform Infrared Spectra (FTIR) were obtained on a Jasco FT/IR 4200 Spectrometer equipped with a diamond attenuated total reflectance accessory (Diamond MIRacle ATR, Pike Technologies, US). A total number of 3 pieces per sterilization technique or control and 3 points on each scaffold were analysed. Spectra were produced from an average of 20 scans at 4 cm^−1^ resolution over a range of 600 cm^−1^ to 4000 cm^−1^ wave numbers. A background scan was performed prior to each sample measurement.

#### Gel Permeation Chromatography (GPC)

Average molecular weight of all 4 PUs was determined by gel permeation chromatography (GPC). The solvent phase was dimethylacetamide (DMAc) with 2% LiBr serving as a stabiliser. The sample concentration was 2 mg/mL and the injection volume was 150 μL. The flow rate was set at 1 mL/min. The number and weight average molecular weights (*M*_*n*_ and *M*_*w*_, respectively) were determined from the retention time using a calibration curve generated with polystyrene standards.

#### Differential Scanning Calorimetry (DSC)

A Q2000 DSC (TA instruments LLC) was used to study glass transition (*T*_*g*_), crystallization and melting behaviour of PUs. An indium standard (*T*_*m*_ = 156.6 °C and Δ*H* = 28.72 J/g) was used to calibrate the instrument and ensure accuracy and reliability of the obtained data. Approximately 4–8 mg of polymer sample were loaded into aluminium Tzero pans and hermetically sealed. An empty aluminium pan, matched in weight to the sample pan, was used as a reference. Experiments were performed at heating rates of 10 °C/min from −80 °C to 200 °C for conventional DSC. Melting temperatures (*T*_*m*_) and heat of fusion (Δ*H*) were determined from DSC scans. Endothermic peak temperatures were taken as *T*_*m*_, and percentage crystallinity was calculated using the following equation:


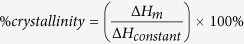


where Δ*H*_*m*_ is the enthalpy of fusion of the sample and Δ*H*_*constant*_ is the enthalpy of fusion of a 100% crystalline PCL reference (136 J/g)^55^,^56^.

#### X-Ray Diffraction (XRD) Crystallography

X-ray diffraction (XRD) data were recorded with a Rigaku MiniFlex 600 (Rigaku, USA) using a Cu Kα X-ray source (λ = 1.5418Å) operated at 15 mA and 40 kV. Measurements were taken from 0 to 40° on the 2*θ* scale at 0.01° step size.

#### Scanning Electron Microscopy (SEM) Evaluation of Surface Changes

Differences in scaffold surface microscopic appearances were evaluated using SEM digital photography at magnifications of ×100, ×500 and ×1000. Degraded scaffolds were washed with PBS and air-dried for a minimum of 3 days. Then, samples were mounted on aluminium stubs and sputter-coated with gold using an SC500 (EMScope) for electrical conductance. Photographs were taken using a Philips 501 scanning electron microscope.

#### Static Contact Angel Measurement to Determine Surface Wettability

Contact angle (*θ*) measurements were obtained using a goniometer (EasyDrop DSA20E, Kruss, Germany) equipped with a digital camera and image analysis software (*DSA1* version 1.80, Kruss, Germany). De-ionized water was used as the wetting liquid which was deposited onto the samples using an automated syringe. Sessile drop method was used to analyse contact angles of the air-water-substrate interface of control and sterilised cast samples which were measured three times in three samples of every group.

#### Tensiometry

Tensile stress-strain properties were assessed according to British Standards (BS ISO 37:2005). Cast or coagulated POSS-PCLU samples were cut into dumbbell-shaped pieces type 3 (shaft length 20 mm, width 4 mm, n = 5) using a cutting press (Wallace Instruments, Surrey, UK). Thickness was measured using a digital electronic outside micrometer. Uniaxial tension was applied to either ends of the scaffolds until failure using an Instron-5565 tensile tester (Instron Ltd., Bucks, UK) equipped with a 500 N load, pneumatic grips with 1 kN capacity and at a rate of 100 mm/min. Bluehill software was used to analyse the scaffold’s tensile strengths at room temperature. Stress (MPa) was calculated by dividing the force generated during stretching by the initial cross-sectional area. Strain was calculated as the ratio of the change in length in reference to the original sample length (%).

### Cell Viability

#### Preparation of Medium Containing Degradation Products

Throughout the polymer degradation study, the degradation medium was renewed once a week to replenish enzymatic or peroxide activity. The old medium was collected and stored in −80 °C until further use. Prior to starting extract cytotoxicity studies, the media containing polymer leachables were filter sterilized and mixed with fresh medium at a range of concentrations (0 – control, 10, 50, and 70% v/v).

#### *In Vitro* Cytocompatibility of Leachables

The cytocompatibility of any products released by the degradation of POSS-PCLU with varying percentage hard segments and PCLU-24 was assessed according to ISO standard 10993-5 using HDFa. Cells were seeded onto tissue culture plastic of 24-well plates at low densities of 2 × 10^4^ cells/cm^2^ in complete culture medium. After 24 h, cells were microscopically assessed and subconfluency was verified. Medium was removed and replaced with fresh medium containing PU leachables at different concentrations. 10% dimethylsulfoxide (DMSO) served as the positive control^57^,^58^ and cells grown in complete medium with no added leachables were the negative control. Cytotoxicity was defined as cell viability below 70% of the negative control as defined by the ISO standard 10993-5. At 6 h, 24 h, 72 h and 7 days, cell viability and proliferative capacity were assessed using AlamarBlue^®^ cell proliferation assay.

### *In vivo* Animal model evaluation

All experiments were conducted under a project license approved by the UK Home Office in accordance with the Animals (Scientific Procedures) Act 1986. 26 male healthy Sprague-Dawley rats weighing 270–300 g were acclimatized for 1 week prior to any experimental procedure. All animals were housed in special facilities with controlled temperatures (24 ± 1 °C) and a 12 h light/dark cycle. Animals had liberal access to water and a standard rat diet.

#### Scaffold Implantation into Animals

Eight weeks old rats were anaesthetised with isofluorane and a dorsal midline incision was created. Two porous scaffolds were implanted subcutaneously on either side of the midline. Round scaffolds (1 mm thickness, 16 mm diameter) were obtained using a cutting press, sterilized in 70% ethanol and then washed 3 times with sterile PBS before implantation. One central suture restricted disc migration and folding in on itself.

#### *In Vivo* evaluation of microcirculation and Oxygenation within the Scaffolds

At 4, 8, and 12 weeks, animals were anaesthetized using isofluorane and scaffolds were exposed. Real-time microvascular blood flow and scaffold oxygenation were measured using Laser Doppler imager (Moor Instruments, Devon, UK) and Visisens^TM^ oxygen sensor (PreSens, Regensburg, Germany), respectively.

#### Scaffold and Organ Harvest and Preparation for Histology

After assessment of scaffold blood flow and oxygenation, animals were sacrificed humanely and both scaffolds and end organs (liver, spleen, brain, lung, and kidney) were harvested and placed in 4% paraformaldehyde. Fixed samples were processed conventionally to produce 4 mm thin paraffin sections and stained with haematoxylin and eosin (H&E). Sections were analysed by a consultant histopathologist blinded to the study objectives.

#### Immunostaining of Explanted Scaffolds

Vascular infiltration of scaffolds was determined using goat polyclonal anti-rat CD31 (Santa Cruz) which identifies endothelial cells. Formalin fixed samples were cut at 5 mm, dewaxed, rehydrated and antigen retrieval performed for CD31 by microwaving in 500 mL Tris and EDTA for 3 min. Sections were then incubated in 5% H_2_O_2_ in methanol to block endogenous peroxidase and incubated with the primary antibodies with normal rabbit or horse serum for 1 h at room temperature at a 1:300 dilution. The sections were then washed in PBS and incubated with biotinylated secondary stage antibody (rabbit anti goat) for 30 min and the reaction product was visualised by DAB. Sections were counterstained using haematoxylin.

### Data analysis and statistics

All data are presented as mean ± standard deviation (SD). Experiments were repeated 6 times unless stated otherwise. Data comparisons were carried out by one-way ANOVA analysis of variance. Significant differences between experimental groups were determined using Bonferroni’s test of multiple comparisons or Dunn’s multiple comparison post-test in the case of surface roughness analysis. A *p*-value of ≤0.05 was considered statistically significant.

## Additional Information

**How to cite this article**: Yildirimer, L. *et al.* Controllable degradation kinetics of POSS nanoparticle-integrated poly(ε-caprolactone urea)urethane elastomers for tissue engineering applications. *Sci. Rep.* **5**, 15040; doi: 10.1038/srep15040 (2015).

## Supplementary Material

Supplementary Information

## Figures and Tables

**Table 1:** Composition of the 4 different polyurethanes.

	POSS content (%)	Diisocyanate content (%)	Polyol	Chain extender
PCLU-24	0	HMDI (24%)	1-Butanol	Ethylenediamine/diethylamine
POSS-PCLU-24	2	HMDI (24%)	1-Butanol	Ethylenediamine/diethylamine
POSS-PCLU-28	2	HMDI (28%)	1-Butanol	Ethylenediamine/diethylamine
POSS-PCLU-33	2	HMDI (33%)	1-Butanol	Ethylenediamine/diethylamine

*Key:* POSS, polyhedral oligomeric silsesquioxane; PCLU, poly(ε-caprolactone urea)urethane; HMDI, dicyclohexylmethane diisocyanate.

**Table 2:** Polymer characteristics of non-degraded (control) polyurethane films and films incubated in degradation media for a period of 6 months.

	Degradation buffer	*T*_*g*_ (°C)^a^		*T*_*m*_ (°C)^a^		Tensile strength (MPa)	Breaking strain (%)	Initial modulus (MPa)	*χ*(%)^a^	*M*_*n*_ (x 10^4^) g/mol^b^	*M*_*w*_*/M*_*n*_^b^	Contact angle (°)
		Soft s.	Hard s.	Soft s.	Hard s.							
PCLU-24	Control	−54.8	51.1	—	—	11.6	342.7	8.1	—	11.5	1.7	86.4
	PBS	−55.4	41.7	—	—	15.4	444.6	5.8	—	11.9	1.5	91.5
	Lipase	−50.4	14.8	38.8	47.7	—	—	—	17.2	1.0	2.0	—
	Collagenase	−55.1	—	43.7	—	12.6	495.9	28.1*	0.4	10.4	1.7	91.7
	CE	−55.9	0.0	41.4	—	11.5	482.8	11.6	2.1	7.6	1.8	87.6
	H_2_O_2_ + CoCl_2_	−23.5	—	46.4	—	—	—	—	37.3	0.6	1.9	—
POSS-PCLU-24	Control	−52.9	40.8	—	—	16.2	456.1	6.3	—	11.9	1.4	105.2
	PBS	−56.7	39.4	—	—	12.5	439.8	5.4	—	10.4	1.5	97.3
	Lipase	−49.4	14.4	39.5	51.8	—	—	—	19.4	1.0	1.9	—
	Collagenase	−54.9	—	41.8	—	19.1	515.7	21.8*	0.4	9.2	1.6	82.0
	CE	−56.8	—	41.4	—	13.1	441.8	9.1	1.3	7.6	1.7	91.8
	H_2_O_2_ + CoCl_2_	−24.3	—	45.8	52.7	—	—	—	35.8	0.5	1.8	—
POSS-PCLU-28	Control	−55.4	52.5	—	—	14.1	351.6	11.4	—	—	—	102.4
	PBS	−56.3	52.5	—	—	16.6	389.9	8.2	—	—	—	99.2
	Lipase	−51.7	12.8	37.8	48.7	—	—	—	9.3	0.8	1.9	90.0
	Collagenase	−52.6	—	39.4	50.2	8.5	286.6	109.3*	9.0	13.4	2.1	85.6
	CE	−56.5	53.8	—	—	21.4	492.8	8.8	—	—	—	97.1
	H_2_O_2_ + CoCl_2_	−46.8	—	37.7	49.3	—	—	—	6.6	0.7	2.0	—
POSS-PCLU-33	Control	−55.5	54.1	—	—	20.7	355.5	24.1	—	—	—	106.3
	PBS	−56.2	52.1	—	—	20.4	389.6	17.3	—	—	—	91.5
	Lipase	−54.4	11.5	39.5	49.4				3.4	—	—	73.7
	Collagenase	−56.3	—	—	—	24.4	393.6	22.5	—	—	—	84.9
	CE	−56.4	—	—	—	18.3	419.6	13.9	—	—	—	—
	H_2_O_2_ + CoCl_2_	−44.8	—	40.2	51.9	—	—	—	11.8	0.7	1.9	—

*Key:* POSS, polyhedral oligomeric silsesquioxane; PCLU, poly(ε-caprolactone urea)urethane.

^a^*χ* was determined by DSC.

^b^*M*_*n*_ and *M*_*w*_*/M*_*n*_ were determined by GPC. − = Values unavailable as samples did not dissolve in DMAc which suggests insignificant degrees of degradation.

## References

[b1] Guelcher S. A. Biodegradable polyurethanes: synthesis and applications in regenerative medicine. Tissue Eng Part B Rev 14, 3–17, 10.1089/teb.2007.0133 (2008). 18454631

[b2] Guelcher S. A. *et al.* Synthesis of biocompatible segmented polyurethanes from aliphatic diisocyanates and diurea diol chain extenders. Acta Biomater 1, 471–484, 10.1016/j.actbio.2005.02.007 (2005). 16701828

[b3] Chawla A. S., Blais P., Hinberg I. & Johnson D. Degradation of explanted polyurethane cardiac pacing leads and of polyurethane. Biomaterials, artificial cells, and artificial organs 16, 785–800 (1988). 10.3109/10731198809117569 3219417

[b4] Izci Y., Secer H., Akay C. & Gonul E. Initial experience with silver-impregnated polyurethane ventricular catheter for shunting of cerebrospinal fluid in patients with infected hydrocephalus. Neurological research 31, 234–237, 10.1179/174313209X380973 (2009). 19040800

[b5] Rahmani B., Tzamtzis S., Ghanbari H., Burriesci G. & Seifalian A. M. Manufacturing and hydrodynamic assessment of a novel aortic valve made of a new nanocomposite polymer. J Biomech 45, 1205–1211, 10.1016/j.jbiomech.2012.01.046 (2012). 22336198

[b6] Desai M. *et al.* A sutureless aortic stent-graft based on a nitinol scaffold bonded to a compliant nanocomposite polymer is durable for 10 years in a simulated *in vitro* model. J Endovasc Ther 19, 415–427, 10.1583/11-3740mr.1 (2012). 22788896

[b7] Chaloupka K., Motwani M. & Seifalian A. M. Development of a new lacrimal drainage conduit using POSS nanocomposite. Biotechnol Appl Biochem 58, 363–370, 10.1002/bab.53 (2011). 21995539

[b8] Jungebluth P. *et al.* Tracheobronchial transplantation with a stem-cell-seeded bioartificial nanocomposite: a proof-of-concept study. Lancet 378, 1997–2004, 10.1016/s0140-6736(11)61715-7 (2011). 22119609

[b9] Li G., Liu Y., Li D., Zhang L. & Xu K. A comparative study on structure-property elucidation of P3/4HB and PEG-based block polyurethanes. Journal of biomedical materials research. Part A 100, 2319–2329, 10.1002/jbm.a.34173 (2012). 22529029

[b10] Rafiemanzelat F., Fathollahi Zonouz A. & Emtiazi G. Synthesis of new poly(ether-urethane-urea)s based on amino acid cyclopeptide and PEG: study of their environmental degradation. Amino acids 44, 449–459, 10.1007/s00726-012-1353-4 (2013). 22833157

[b11] Li Z., Zhang Z., Liu K. L., Ni X. & Li J. Biodegradable hyperbranched amphiphilic polyurethane multiblock copolymers consisting of poly(propylene glycol), poly(ethylene glycol), and polycaprolactone as *in situ* thermogels. Biomacromolecules 13, 3977–3989, 10.1021/bm3012506 (2012). 23167676

[b12] Tokiwa Y., Calabia B. P., Ugwu C. U. & Aiba S. Biodegradability of plastics. International journal of molecular sciences 10, 3722–3742, 10.3390/ijms10093722 (2009). 19865515 PMC2769161

[b13] Tokiwa Y. & Suzuki T. Hydrolysis of polyesters by Rhizopus delemar lipase. Agric. Biol. Chem. 42, 1071–1072 (1978).

[b14] Iwata T. & Doi Y. Morphology and enzymatic degradation of poly(L-lactic acid) single crystals. Macromolecules 31, 2461–2467 (1998).

[b15] Gu X., Wu J. & Mather P. T. Polyhedral oligomeric silsesquioxane (POSS) suppresses enzymatic degradation of PCL-based polyurethanes. Biomacromolecules. 12, 3066–3077 (2011). 21675705 10.1021/bm2006938

[b16] Labow R. S., Tang Y., McCloskey C. B. & Santerre J. P. The effect of oxidation on the enzyme-catalyzed hydrolytic biodegradation of poly(urethane)s. Journal of biomaterials science. Polymer edition 13, 651–665 (2002). 12182550 10.1163/156856202320269148

[b17] Williams D. F. & Zhong S. P. Biodeterioration/biodegradation of polymeric medical devices *in situ*. International Biodeterioration & Biodegradation 34, 95–130 (1994).

[b18] Lee K. H. & Chu C. C. The role of superoxide ions in the degradation of synthetic absorbable sutures. Journal of biomedical materials research 49, 25–35 (2000). 10559743 10.1002/(sici)1097-4636(200001)49:1<25::aid-jbm4>3.0.co;2-i

[b19] Sutherland K., Mahoney J. R., Coury A. J. & Eaton J. W. Degradation of biomaterials by phagocyte-derived oxidants. The Journal of clinical investigation 92, 2360–2367, 10.1172/JCI116841 (1993). 8227352 PMC288418

[b20] Santerre J. P., Labow R. S., Duguay D. G., Erfle D. & Adams G. A. Biodegradation evaluation of polyether and polyester-urethanes with oxidative and hydrolytic enzymes. Journal of biomedical materials research 28, 1187–1199, 10.1002/jbm.820281009 (1994). 7829548

[b21] Tang Y. W., Labow R. S. & Santerre J. P. Enzyme-induced biodegradation of polycarbonate polyurethanes: dependence on hard-segment concentration. Journal of biomedical materials research 56, 516–528 (2001). 11400129 10.1002/1097-4636(20010915)56:4<516::aid-jbm1123>3.0.co;2-b

[b22] Santerre J. P. & Labow R. S. The effect of hard segment size on the hydrolytic stability of polyether-urea-urethanes when exposed to cholesterol esterase. Journal of biomedical materials research 36, 223–232 (1997). 9261684 10.1002/(sici)1097-4636(199708)36:2<223::aid-jbm11>3.0.co;2-h

[b23] Li F. K. *et al.* Studies on Thermally Stimulated Shape-Memory Effect of Segmented Polyurethanes. Journal of Applied Polymer Science 64, 1511–1516 (1997).

[b24] Shoae-Hassani A. *et al.* lambda Phage Nanobioparticle Expressing Apoptin Efficiently Suppress Human Breast Carcinoma Tumor Growth *In Vivo*. PLoS One 8, e79907, 10.1371/journal.pone.0079907 (2013). 24278212 PMC3838365

[b25] Shoae-Hassani A. *et al.* Endometrial stem cell differentiation into smooth muscle cell: a novel approach for bladder tissue engineering in women. BJU Int 112, 854–863, 10.1111/bju.12195 (2013). 24028767

[b26] Guasti L., Vagaska B., Bulstrode N. W., Seifalian A. M. & Ferretti P. Chondrogenic differentiation of adipose tissue-derived stem cells within nanocaged POSS-PCU scaffolds: A new tool for nanomedicine. Nanomedicine, 10.1016/j.nano.2013.08.006 (2013). 24008020

[b27] Uhrich K. E., Cannizzaro S. M., Langer R. S. & Shakesheff K. M. Polymeric systems for controlled drug release. Chem Rev 99, 3181–3198 (1999). 11749514 10.1021/cr940351u

[b28] Gao Y., Eguchi A., Kakehi K. & Lee Y. C. Efficient preparation of glycoclusters from silsesquioxanes. Organic letters 6, 3457–3460, 10.1021/ol040043a (2004). 15387522

[b29] Christenson E. M., Patel S., Anderson J. M. & Hiltner A. Enzymatic degradation of poly(ether urethane) and poly(carbonate urethane) by cholesterol esterase. Biomaterials 27, 3920–3926, 10.1016/j.biomaterials.2006.03.012 (2006). 16600363

[b30] Peng L. H. *et al.* Transplantation of bone-marrow-derived mesenchymal and epidermal stem cells contribute to wound healing with different regenerative features. Cell and tissue research 352, 573–583, 10.1007/s00441-013-1609-7 (2013). 23568655

[b31] Göpferich A. Mechanisms of polymer degradation and erosion. Biomaterials 17, 103–114 (1996). 8624387 10.1016/0142-9612(96)85755-3

[b32] Schutzius T. M., Bayer I. S., Jursich G. M., Das A. & Megaridis C. M. Superhydrophobic-superhydrophilic binary micropatterns by localized thermal treatment of polyhedral oligomeric silsesquioxane (POSS)-silica films. Nanoscale 4, 5378–5385, 10.1039/c2nr30979c (2012). 22820974

[b33] Sung C. S. P., Hu C. B. & Wu C. S. Properties of Segmented Poly(rethaneureas) Based on 2,4-Toluene Diisocyanate. 1. Thermal Transitions, X-ray Studies, and Comparison with Segmented Poly(urethanes). Macromolecules 13, 111–111 (1980).

[b34] O'Sickey M. J., Lawrey B. D. & Wilkes G. L. Structure-Property Relationships of Poly(urethane urea)s with Ultra-low Monol Content Poly(propylene glycol) Soft Segments. 1. Influence of Soft Segment Molecular Weight and Hard Segment Content. Journal of Applied Polymer Science 84, 229–229 (2002).

[b35] Korley L. T. J., Pate B. D., Thomas E. L. & Hammond P. T. Effect of the degree of soft and hard segment ordering on the morphology and mechanical behavior of semicrystalline segmented polyurethanes. Polymer 47, 3073–3082, 10.1016/j.polymer.2006.02.093 (2006).

[b36] Skarja G. A. & Woodhouse K. A. Synthesis and characterization of degradable polyurethane elastomers containing an amino acid-based chain extender. Journal of Biomaterials Science, Polymer Edition 9, 271–295, 10.1163/156856298X00659 (1998). 9556762

[b37] Wang G. B., Labow R. S. & Santerre J. P. Biodegradation of a poly(ester)urea-urethane by cholesterol esterase: isolation and identification of principal biodegradation products. Journal of biomedical materials research 36, 407–417 (1997). 9260112 10.1002/(sici)1097-4636(19970905)36:3<407::aid-jbm16>3.0.co;2-a

[b38] Shibayama M., Kawauchi T., Kotani T., Nomura S. & Matsuda T. Structure and properties of fatigued segmented poly(urethaneurea)s I. Segment orientation mechanism due to fatigue. Polym. J. 18, 719–733 (1986).

[b39] Gaisford S. & Saunders M. Essentials of Pharmaceutical Preformulation. (John Wiley & Sons, 2013).

[b40] Okkema A. Z. & Cooper S. L. Effect of carboxylate and/or sulphonate ion incorporation on the physical and blood-contacting properties of a polyetherurethane. Biomaterials 12, 668–676 (1991). 1742412 10.1016/0142-9612(91)90115-q

[b41] Szycher M., Poirier V. L. & Dempsey D. J. Development of an aliphatic biomedical-grade polyurethane elastomer. J Elastom Plast. 81.95–81.95 (1983).

[b42] Shibayama M., Suetsugu M., Sakurai S., Yamamoto T. & Nomura S. Structure characterization of polyurethanes containing poly(dimethylsiloxane). Macromolecules 24, 6254–6262, 10.1021/ma00023a031 (1991).

[b43] Ostwald W. Z. Studien über die Bildung und Umwandlung fester Körper. Zeitschrift Für Physikalische Chemie 22, 289–330 (1897).

[b44] Engelberg I. & Kohn J. Physico-mechanical properties of degradable polymers used in medical applications: a comparative study. Biomaterials 12, 292–304 (1991). 1649646 10.1016/0142-9612(91)90037-b

[b45] Chan-Chan L. H. *et al.* Degradation studies on segmented polyurethanes prepared with HMDI, PCL and different chain extenders. Acta Biomater 6, 2035–2044, 10.1016/j.actbio.2009.12.010 (2010). 20004749

[b46] Lee W. C. & Chu I. M. Preparation and degradation behavior of polyanhydrides nanoparticles. J Biomed Mater Res B Appl Biomater 84, 138–146, 10.1002/jbm.b.30854 (2008). 17474078

[b47] Tatai L. *et al.* Thermoplastic biodegradable polyurethanes: the effect of chain extender structure on properties and *in-vitro* degradation. Biomaterials 28, 5407–5417, 10.1016/j.biomaterials.2007.08.035 (2007). 17915310

[b48] Bernacca G. M., O'Connor B., Williams D. F. & Wheatley D. J. Hydrodynamic function of polyurethane prosthetic heart valves: influences of Young’s modulus and leaflet thickness. Biomaterials 23, 45–50 (2002). 11762853 10.1016/s0142-9612(01)00077-1

[b49] Rizvi S. B. *et al.* A novel POSS-coated quantum dot for biological application. Int J Nanomedicine 7, 3915–3927, 10.2147/ijn.s28577 (2012). 22915843 PMC3418109

[b50] Hafeman A. E. *et al.* Characterization of the degradation mechanisms of lysine-derived aliphatic poly(ester urethane) scaffolds. Biomaterials 32, 419–429, 10.1016/j.biomaterials.2010.08.108 (2011). 20864156 PMC2997347

[b51] Ahmed M., Punshon G., Darbyshire A. & Seifalian A. M. Effects of sterilization treatments on bulk and surface properties of nanocomposite biomaterials. J Biomed Mater Res B Appl Biomater 101, 1182–1190, 10.1002/jbm.b.32928 (2013). 24039066 PMC4228764

[b52] Taylor M. S., Daniels A. U., Andriano K. P. & Heller J. Six bioabsorbable polymers: *in vitro* acute toxicity of accumulated degradation products. Journal of applied biomaterials: an official journal of the Society for Biomaterials 5, 151–157, 10.1002/jab.770050208 (1994). 10147175

[b53] Marcos-Fernández A., Abraham G. A., Valentín J. L. & Román J. S. Synthesis and characterization of biodegradable non-toxic poly(ester-urethane-urea)s based on poly(ε-caprolactone) and amino acid derivatives. Polymer 47, 785–798 (2006).

[b54] Kannan R. Y. *et al.* The antithrombogenic potential of a polyhedral oligomeric silsesquioxane (POSS) nanocomposite. Biomacromolecules 7, 215–223, 10.1021/bm050590z (2006). 16398518

[b55] Crescenzi V., Manzini G., Calzolari G. & Borri C. Thermodynamics of fusion of poly-ß-propiolactone and poly-ε-caprolactone. Comparative analysis of the melting of aliphatic polylactone and polyester chains. Eur Polym 8, 449–463 (1972).

[b56] Heijkants R. G. J. C. *et al.* Uncatalyzed synthesis, thermal and mechanical properties of polyurethanes based on poly(epsilon-caprolactone) and 1,4-butane diisocyanate with uniform hard segment. Biomaterials 26, 4219–4228, 10.1016/j.biomaterials.2004.11.005 (2005). 15683644

[b57] Bae S. H. *et al.* *In vitro* biocompatibility of various polymer-based microelectrode arrays for retinal prosthesis. Investigative ophthalmology & visual science 53, 2653–2657, 10.1167/iovs.11-9341 (2012). 22427592

[b58] Hua K. *et al.* Translational study between structure and biological response of nanocellulose from wood and green algae. RSC Advances 4, 2892–2903 (2014).

