# Supplementary material for: Controllable degradation kinetics of POSS nanoparticle-integrated poly(ε-caprolactone urea)urethane elastomers for tissue engineering applications
Source: Sci Rep. 2015 Oct 14;5:15040. doi: 10.1038/srep15040 (PMC4604490; doi:10.1038/srep15040)
Supplement: Supplementary Information [file srep15040-s1.doc]

**Supplementary information**

**Controllable degradation kinetics of POSS nanoparticle-integrated poly(ε-caprolactone urea)urethane elastomers for tissue engineering applications**

Lara Yildirimer^1^, Asma Buanz^2^, Simon Gaisford^2^, Edward L. Malins^3^, C. Remzi Becer^4^, Naiem Moiemen^5,6^, Gary M. Reynolds^7^ Alexander M. Seifalian^1,8*^

*^1^Centre for Nanotechnology & Regenerative Medicine, UCL Division of Surgery & Interventional Science, University College London, ^2^UCL School of Pharmacy, University College London, 29-39 Brunswick Square, London WC1N 1AX, UK, ^3^Department of Chemistry, University of Warwick, CV4 7AL, Coventry, UK, ^4^School of Engineering and Materials Science, Queen Mary University of London, E1 4NS, London, UK, ^5^Department of Burns and Plastic Surgery, University Hospitals Birmingham NHS Foundation Trust, Queen Elizabeth Hospital, Birmingham, United Kingdom, ^6^*[*The Healing Foundation Children's Burns Research Centre*](http://www.bristol.ac.uk/social-community-medicine/childrens-burns/)*, University of Bristol, Senate House, Tyndall Avenue, Bristol, BS8 1TH, UK, ^7^Centre for Liver Research & NIHR BRU, Queen Elizabeth Hospital & University of Birmingham, ^8^Royal Free Hampstead NHS Trust Hospital, Pond Street, NW3 2QG, London, UK.*


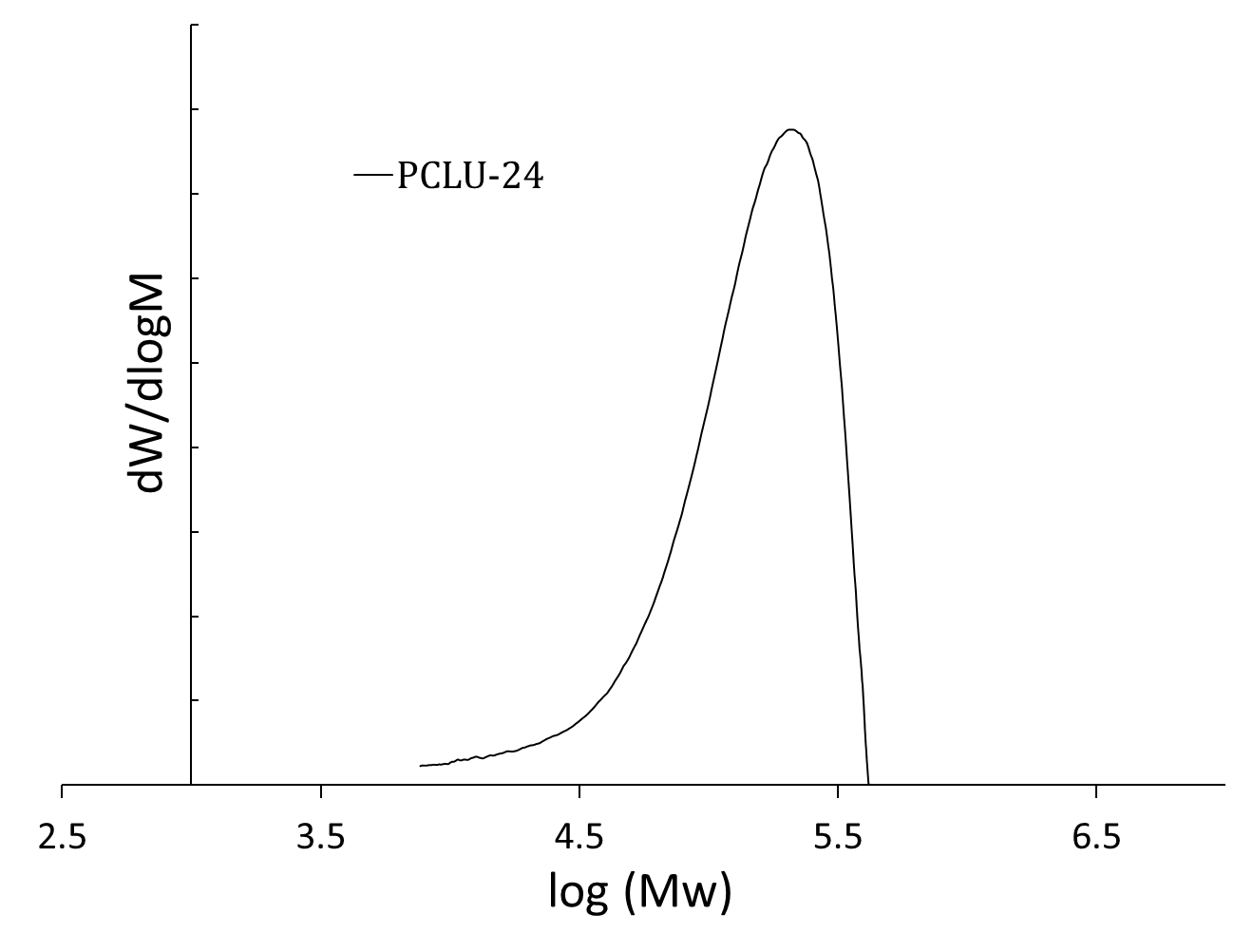


**Supplementary Figure 1.** Molecular weight distribution curve of non-degraded PCLU-24.


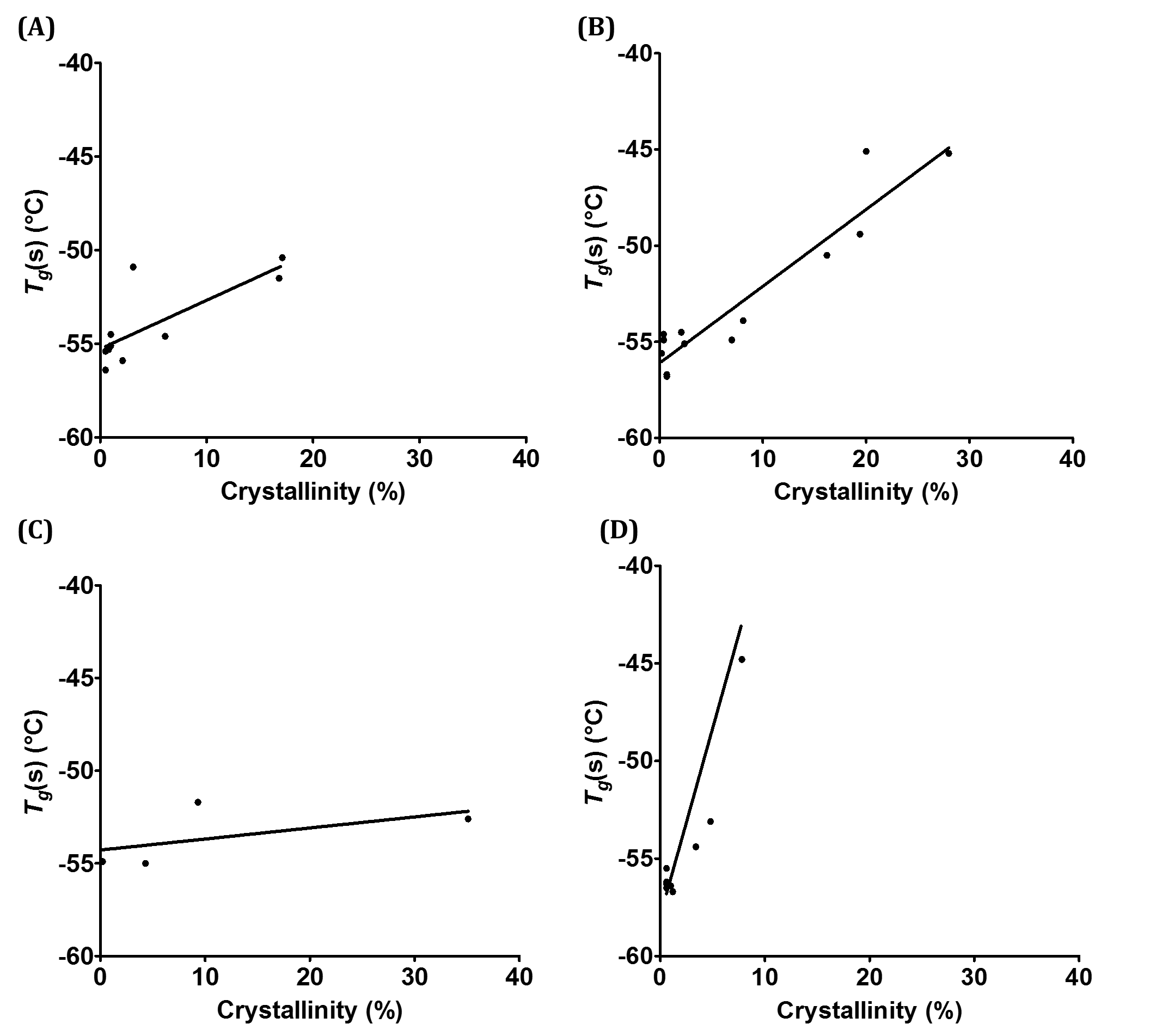


**Supplementary Figure 2.** Soft segment glass transition temperatures (Tg(s)) as a function of percentage crystallinity of degraded (A) PCLU-24, (B) POSS-PCLU-24, (C) POSS-PCLU-28 and (D) POSS-PCLU-33 polymers.

**Supplementary Figure 3.** The normalized step height of the specific heat at Tg as a function of crystallinity.


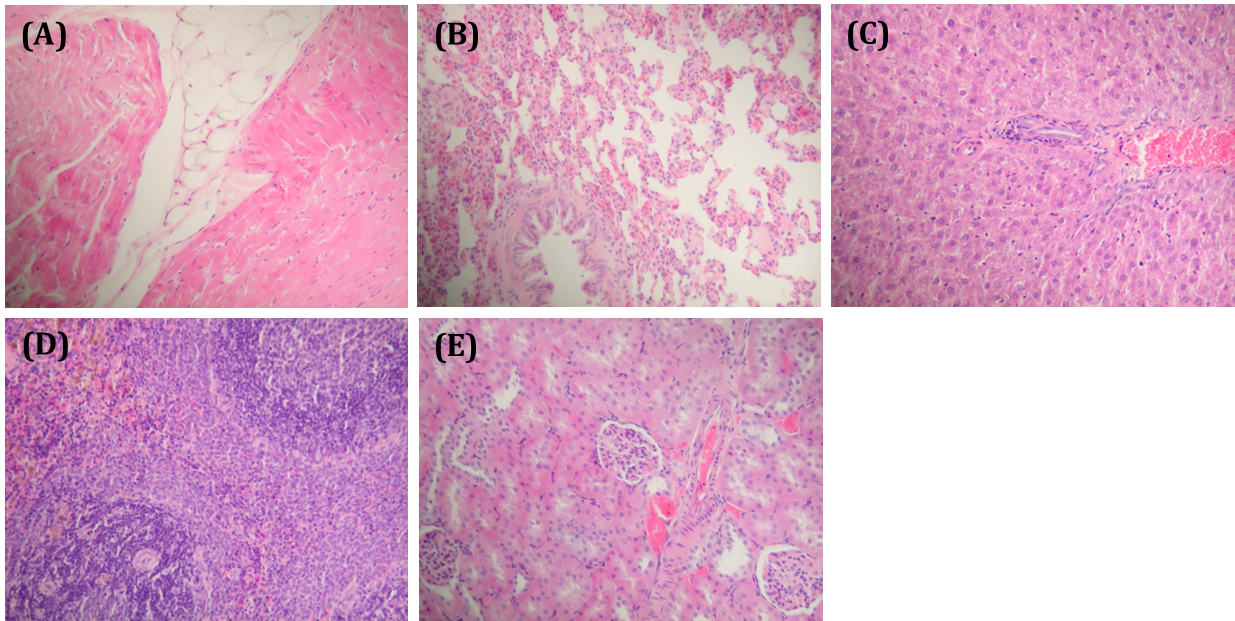


**Supplementary Figure 4.** Representative haematoxylin & eosin stains of in vivo response to implanted scaffolds. Sections of heart (A), lung (B), liver (C), spleen (D) and kidney (E) tissues appear normal.
